# A window in time for β-cell regeneration

**DOI:** 10.1101/gad.345769.120

**Published:** 2020-12-01

**Authors:** Benjamin J. Weidemann, Joseph Bass

**Affiliations:** Department of Medicine, Division of Endocrinology, Metabolism, and Molecular Medicine, Northwestern University Feinberg School of Medicine, Chicago, Illinois 60611, USA

**Keywords:** circadian clockwork, pancreatic α and β cells, Insulin-rtTA/TET-DTA mouse model, diabetes, glucose metabolism, β-cell proliferation, β-cell regeneration

## Abstract

In vivo regeneration of β cells provides hope for self-renewal of functional insulin-secreting cells following β-cell failure, a historically fatal condition now sustainable only by administration of exogenous insulin. Despite advances in the treatment of diabetes mellitus, the path toward endogenous renewal of β-cell populations has remained elusive. Intensive efforts have focused on elucidating pancreatic transcriptional programs that can drive the division and (*trans*-)differentiation of non-β cells to produce insulin. A surprise has been the identification of an essential role of the molecular circadian clock in the regulation of competent insulin-producing β cells. In this issue of *Genes & Development*, work by Petrenko and colleagues (pp. 1650–1665) now shows a requirement for the intrinsic clock in the regenerative capacity of insulin-producing cells following genetic ablation of β cells. These studies raise the possibility that enhancing core clock activity may provide an adjuvant in cell replacement therapies.

## Abstract (precis)

This Outlook discusses Petrenko et al.’s finding reporting a requirement for the intrinsic clock in the regenerative capacity of insulin-producing cells following genetic ablation of β cells.

A transformation in understanding the molecular basis for biological timing came from the genetic analyses of 24-h circadian sleep/wake rhythms in *Drosophila* and mice (circadian from circa diem, about a day) (Cederroth et al. 2019). Positional cloning revealed the clock to be encoded by an autoregulatory transcriptional feedback loop in which the forward limb (circadian locomotor output cycles kaput [CLOCK]/brain and muscle aryl hydrocarbon receptor nuclear translocator-like 1 [BMAL1]) induces the expression of repressors (period [PER]/cryptochrome [CRY]/reverse c-erbA [REV-ERB]) that provide feedback to inhibit the forward limb in a cycle that repeats itself every 24 h. A paradigm shift came with the recognition that clocks are present not only within the brain but also within nearly all animal cells, indicating that circadian pathways regulate both behavior and systemic physiology. Genetic evidence that clocks exert ubiquitous control over tissue function emerged following the observation that disruption of the clock in the islet of adult mice leads to obesity and hypoinsulinemic diabetes—a hallmark of β-cell failure in human diabetes (Perelis et al. 2015). Although molecular clocks have been shown to affect cell cycle and development in a variety of tissues, the mechanism and function of clocks in mammalian β-cell fate and regenerative capacity is not known.

Thorel et al. (2010) established a powerful genetic model to examine β-cell regeneration by showing that seemingly terminally differentiated adult pancreatic cells could be converted to insulin-secreting cells in vivo following diphtheria-induced β-cell ablation during adulthood. New insulin-secreting cells were observed within islets once devoid of β cells, and genetic tracing of pancreatic cell types revealed that a substantial proportion of these insulin-secreting cells arose from either non-β cells or β-cell precursors. Using a similar model, in which doxycycline-induced expression of diphtheria toxin A (DTA) triggers β-cell destruction, Petrenko et al. (2020) inquired whether the circadian clock might be required for the regeneration of functional insulin-secreting cells by comparing responses to doxycycline following knockout of the gene encoding the core clock activator BMAL1. Their results establish a central role of the molecular clock in β-cell regenerative capacity (Fig. 1).

**Figure 1.**
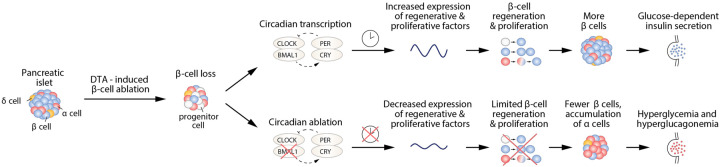
β-Cell ablation triggers clock-dependent β-cell regeneration. In this issue of *Genes & Development*, Petrenko et al. (2020) administer doxycycline (DOX) to adult transgenic mice, triggering diphtheria toxin (DTA) expression within β cells, causing abrupt β-cell destruction. This is followed by DOX washout, leading to regeneration from progenitor endocrine cells. (*Top*) RNA profiling across the day/night cycle in DTA-treated mice revealed robust rhythms in circadian and regenerative gene networks within a renewing β-cell population. (*Bottom*) Unexpectedly, in DTA-ablated mice that were nullizygous for the core clock gene *Bmal1*, there was abrogation of β-cell regeneration and accumulation of glucagon-producing cells within the islet. These observations reveal a requirement for the circadian transcription factor pathway in islet regeneration following massive β-cell loss.

Following DTA ablation of β cells in mice harboring a functional clock, the investigators first demonstrate that regenerative cells exhibit robust genome-wide transcription including oscillation of RNAs encoding the core clock components retinoic acid receptor-related orphan receptor γ (RORγ), BMAL1, and CRY1, as well as cell cycle machinery, including Ki-67, a marker of proliferation. The regenerative program elicited by destruction of β cells suggests that resident cells that do not secrete insulin or are insensitive to DTA are set on a path toward an insulin-secreting cell phenotype, and this path likely requires diurnal patterns of transcription in tandem with induction of the core β-cell machinery. While previous studies showed that activation of β-cell transcription factors (i.e. neurogenin-3 [NGN3] pancreatic and duodenal homeobox 1 [*Pdx1*], and v-maf avian musculoaponeurotic fibrosarcoma A [MAFA]) (Zhou et al. 2008) is sufficient to drive glucose-responsive insulin secretion from extraislet exocrine cells, stem cells and transdifferentiated cells are often not glucose-responsive or simultaneously express non-β-cell hormones alongside insulin. Nonetheless, the regenerative machinery required to restore β-cell function in late stage diabetes remains mysterious. Prior studies suggest that circadian transcriptional oscillation represents an early hallmark of the functional β cell (Rakshit et al. 2018). Furthermore, exposure of stem cells to a combination of forskolin, arginine, glucose, and insulin to induce synchronous circadian transcriptional rhythms enhances maturation into insulin-secreting cells (Alvarez-Dominguez et al. 2020). Collectively, this study from Petrenko et al. (2020) supports the idea that circadian transcriptional rhythms may be a feature of healthy insulin-secreting β cells in the context of regeneration.

A second surprise is the finding that genetic ablation of BMAL1 wipes out the capacity for β cells to regenerate following DTA expression. This implicates a functional role of the core clock transcription factor pathway in β-cell differentiation and/or maturation. While clock transcription factors have been shown to regulate adult life α- and β-cell hormone secretion (Marcheva et al. 2010), the present results indicate a role in endocrine cell fate. The third key observation is that ablation of insulin-producing cells in *Bmal1* nullizygous animals led to accumulation of glucagon-positive α cells, indicating a requirement for the molecular clock in the interconversion of α- and β-cell types following β-cell ablation. Collectively, these experiments are important in being the “first in kind” proof that the core clock and rhythmic processes are involved in β-cell regeneration—a finding with implications across multiple organs where clock expression may modulate regenerative pathways throughout life and responses to environmental challenges.

Transcriptomic studies identify gene networks that are likely downstream from core clock (or PAR-ZIP) factors, such as the forkhead box protein M1 (FOXM1) and translation control factors such as the mammalian target of rapamycin (mTOR) and eukaryotic initiation factor 2 (eIF2). Although there remains debate concerning the biology of β-cell loss and replacement in health and disease states, the findings here open the field to future mechanistic examination. Coordination of clock control of chromatin and tissue-specific transcription factors likely plays a role in regeneration, although elucidating the molecular programs linking circadian and regenerative pathways will be critical moving forward. Rhythmic chromatin activation by the clock may play a role in β-cell regeneration, involving collaboration of clock activators (CLOCK/BMAL1) and associated histone acetyltransferase complexes with factors such as FOXM1. Alternatively, the timed recruitment of clock repressors (PER/CRY/REVERB) and associated histone deacetylases may play a key role in progression from α- to β-cell fate. It remains unclear whether clock-dependent regeneration is driven by activation or repression of chromatin.

While the present studies focus on loss-of-function analyses, a remaining question is whether gain of clock oscillation may augment regenerative capacity. As demonstrated here and in prior studies, regeneration of β-cell mass occurs in the adult rodent; however, unlike rodents, there is limited evidence for the in vivo regeneration of pancreatic β-cell population in humans (Menge et al. 2008). This raises an intriguing possibility that endogenous pathways, such as the highly conserved circadian timing system, could serve as a therapeutic target to unlock human islet regenerative potential, either in stem cell allografts or through in vivo therapeutics. Manipulation of the circadian clock transcription pathway may enhance the capacity to regenerate β cells in the setting of diabetes.
